# The Impact of Preceptorship for Newly Graduated Orthoptists on Clinical Confidence and Attitudes towards Public Health

**DOI:** 10.22599/bioj.248

**Published:** 2022-01-31

**Authors:** Martha Farrelly-Waters, Jignasa Mehta

**Affiliations:** 1 Manchester Foundation Trust, GB; 2 University of Liverpool, GB

**Keywords:** preceptorship, new graduate, orthoptist, mentorship, preceptor, preceptee

## Abstract

**Background:**

Preceptorship has been found to ensure a positive and healthy start to a clinician’s career. Evidence shows increased staff satisfaction and empowerment and decreased medical errors as a result of preceptorship. There is limited literature that includes allied health professionals, particularly new orthoptic graduates. This study aims to: 1) explore the effectiveness of the British and Irish Orthoptic Society (BIOS) preceptorship programme at providing support and confidence in newly graduated orthoptists, 2) explore new graduates’ experience of embedding public health in their clinical practice.

**Methodology:**

Focus groups were organised for mentors and mentees to discuss their experiences with the BIOS preceptorship programme and how it facilitated embedding public health into clinical practice. Constant comparison analysis was used to identify key themes of discussion.

**Findings:**

The preceptorship document promoted structure, reflection, and engagement all of which contributed to effective transition for the mentees. However, document navigation, lack of preceptorship exposure at undergraduate level and leadership engagement were potential barriers to using the programme. The programme encouraged public health engagement among new graduates but barriers such as time pressure, lack of experience and patient understanding were challenges that often prevented the adoption of public health skills and behaviours within their practice.

**Conclusion:**

The BIOS preceptorship successfully supports new graduates in their transition into an autonomous practitioner. The programme could be improved by the implementation of a guidance document to assist mentors in their role. Preceptorship engagement could be improved by increasing exposure to undergraduate orthoptic students and departments alike.

## Introduction

A preceptorship is a period of time where a newly graduated clinician receives support from a mentor in their clinical duties to ensure an efficient and healthy transition from student to autonomous practitioner (Essam N, and Department of Health 2010).

Evidence shows newly qualified allied health professionals (AHPs) often have high expectations of professional competence and these expectations were often unrealistic and taxing on the newly qualified clinicians (Morley et al. 2007). Furthermore, the transition into independent practitioner was reported to cause feelings of vulnerability and loneliness (Bontoft 2007), which emphasises the importance of support during this period. The presence of preceptorship has been shown to improve turnover rates, costs and retention rates while decreasing medical errors among newly qualified nursing staff (Lee et al. 2009). It has also demonstrated increased sense of psychological empowerment and professional autonomy (Watkins, Hart, and Mareno. 2016), hence illustrating the preceptorship to have positive impacts on health organisations, as well as individual confidence and competence.

However, the vague idea of ‘support’ is not enough to ensure an effective transition for new graduates. Bond and Holland (2010) found unstructured support mechanisms to be ineffective, while further evidence found the structure of a preceptorship programme improved competence and confidence in occupational therapists (Maringer 2014). Along with structure being a cornerstone for preceptorship, it has been described as needing to be a nurturing support system (Vance 1982) and the relationship between graduate and mentor should also be a positive one in order to ensure new graduate satisfaction and success (Moore and Cagle 2012). One study reported newly qualified nurses were more likely to feel engaged and satisfied with their work if their preceptor had high levels of authentic leadership (Giallonardo, Wong, and Iwasiw 2010). This suggests an important part of effective preceptorship is engagement and leadership of the preceptor.

Much of the reported literature examines the effect of preceptorship on graduate nurses and nursing careers, with little published evidence on how preceptorship can benefit AHPs. Furthermore, the Public Health strategy for AHPs (2014–19) set out the vision of the future AHP workforce to engage with the public health agenda of prevention. To support this vision, the Council of Deans set out twelve recommendations for Public Health content to be embedded in the pre-registration curricula (Council of Deans 2017). There is a lack of literature in examining the influence of a preceptorship on promoting and embedding public health goals into early career practitioners. The inclusion of Public Health in a preceptorship programme aligns with the AHP Public Health vision of ‘developing the AHP workforce’ (AHP Public Health Strategic Framework 2019–2024). It would give graduates the confidence to embed a preventative and population health approach throughout their clinical work.

In 2018, the British and Irish Orthoptic Society (BIOS) relaunched their preceptorship programme bespoke for newly qualified orthoptists. The programme was revisited following a scoping exercise to review current methods of support for new graduates. A wide variety of support mechanisms were identified ranging from structured tools such as formal preceptorship, personal development meetings and case discussions, to less structured mechanisms such as the use of ‘buddies,’ defined as a fellow orthoptist who would be available for any questions from the new graduate. It was apparent that new graduates across the UK were receiving heterogenous levels of preceptorship resulting in a lack of standardised support to newly qualified graduates.

This scoping exercise contributed to the final preceptorship programme, which was then peer-reviewed and disseminated to Universities and orthoptic departments across the UK. The document required new graduates to record evidence of particular behaviours recognised by BIOS as crucial for effective autonomous working, such as interpersonal and leadership skills, autonomous decision-making with evidence-based practice, reflection, research and audit, and advocacy for public health.

A year after its launch the research team used focus groups to evaluate the effectiveness of the BIOS preceptorship programme. The aims of this qualitative study were to explore:

- The effectiveness of BIOS preceptorship at providing support and developing clinical confidence in newly graduated orthoptists.
- The new graduates’ experience of embedding public health in their clinical practice.

## Methodology

Qualitative focus group interviews were organised to explore the experiences of new graduates and their mentors who completed the programme. A focus group interview, as Patton (1990) states, is a highly efficient data collection technique whereby ‘participants tend to provide checks and balances on each other which weeds out false or extreme views.’ It is also a way of quickly ascertaining some consistent and shared views. Participants (preceptors and preceptees) were recruited following an advert released in the BIOS online newsletter and separated into the following groups:

Group 1: Newly graduate orthoptists within their first two years of working who had completed the BIOS preceptorship.

Group 2: Orthoptists working as a new graduate’s preceptor/mentor who have worked through the BIOS Preceptorship with them.

Group 3: Newly graduated orthoptists who had not completed the BIOS Preceptorship, had completed a preceptorship by a different organisation or who had not had any formal support mechanism.

Henceforth the new graduate preceptees (Group 1) will be referred to as mentees, and the preceptors (Group 2) as mentors. The group interviews were conducted using Microsoft Teams video conference programme. Separate sessions were arranged for each group to avoid participants feeling influenced by their respective mentor/mentee being present. Indicative interview schedules were developed by the research team as a means of prompting the main discussion stimulus, ‘Tell us about the support you experienced in your first year of clinical practice’ for the mentees and ‘Tell us about your experience with the preceptorship programme’ with the mentors. Participants were encouraged to discuss amongst themselves their responses, with little guidance from the researchers JM and MFW. The interview schedules explored participants initial reaction to the programme, areas of the preceptorship that were more/less engaging, reasons for engagement and subsequent impact the programme had on their practice after completion. While the schedules for mentees and mentors were similar, each question was adapted to fit the experience of the mentee and mentor. JM and MFW acted as the moderators for the group and were responsible for facilitating the discussion and prompting the participants to speak. JM facilitated the discussion around the Public Health aspect of the preceptorship.

Ethical approval was granted through the University of Liverpool (reference no. 3558) and the study aligned with the ideals of the Declaration of Helsinki. The interviews were audio- and video-recorded for transcription purposes with the participants’ informed consent.

## Data collection and analysis

The recording was transcribed verbatim by a paid experienced transcriber. Thematic analysis was applied to investigate emerging trends in the discussions. To reduce bias, JM and MFW independently identified themes from the manuscripts that were then categorised under broad headings and supported with direct quotes.

Constant comparison analysis was utilised to identify the themes of discussion. JM and MFW conducted manual line-by-line coding to group together comments of a similar subject matter. These groups were then further categorised into discussion themes. Identified themes were compared internally between the separate focus groups to identify agreements and disagreements between mentors and mentees, as well as externally to identify similarities/differences to current literature.

## Findings and Discussion

Five mentees volunteered to take part with three being available to attend the scheduled date and time of the interview. There was one male and two females, all of whom graduated in 2018. All three mentees were placed in Group 1. Four mentors volunteered to take part in the focus group, of which two were able to attend the scheduled event and placed in Group 2. No volunteers came forward who fulfilled the criteria for Group 3 and therefore only results from Groups 1 and 2 will be reported here.

The themes were broadly grouped into enablers and barriers for uptake of the preceptorship programme followed by the outcomes of having completed the programme from both the mentors and mentees perspectives. The main outcome of the preceptorship was *professional development* discussed by both mentors and mentees and examples of reflection, transition from student to autonomous clinician and career progression were seen throughout their narratives.

Participants were also asked to comment on their experience of implementing public health behaviours into their clinical practice. These behaviours could be anything that promoted public health, for example, Healthy Conversations with patients or promoting disease prevention. Mentees and mentors were able to identify enablers and barriers of public health and identified features of the preceptorship document that supported the implementation process.

### Enablers of the programme

Participants identified the support and leadership from their mentor and the programme structure as aspects that encouraged active engagement and successful completion of the programme.

#### Support/Leadership from mentor

The relationship between mentees and mentors was overall positive, resulting in more effective support for the mentee throughout the programme. Mentor two described the document as a way to ‘let the newly qualified person know that [the department] had an invested interest in their development.’ This is a positive finding as Moore and Cagle (2012) highlight the importance of having a healthy mentor-mentee relationship. In their study, they found physically or emotionally absent mentors resulted in a more negative relationship between them and the new graduate. The mentors agreed the document acted as a catalyst for transition opportunities, and was adaptable to the needs of the mentee:

‘[We] used it very much as a starting point… rather than working through sort of section by section, [the mentees] would bring examples of what had been happening in the clinic, cases that they’d seen or scenarios that they were in and we would sort of work out, well that would fit into this bit or that example would fit into that bit, rather than let’s take this section for the next meeting.’ – *Mentor 2*

‘Similarly, I didn’t stick to it every meeting… we identified what we needed.’ – *Mentor 1*

Mentor 1 reflected on how she believed investment in newly qualified members of staff by means of a preceptorship, could benefit the mentee and mentor:

‘When I think back, I think I actually could have been a better manager to [a previous new graduate] if I’d invested more time in her development at the beginning. And so that’s why I’ve made a conscious effort this time… I think you get more out of your staff when you’ve shown that you’ve invested time in them, and you’re interested in their progression… Maybe if I’d spent more time with her, she might have been more committed to the role.’ *– Mentor 1*

There was a point of interest noted in relation to how the mentors approached their preceptorship meetings as mentor 1 had one mentee completing the programme, while mentor 2 had two mentees at the same time and therefore held group meetings with the mentees together. Mentor 2 found this experience very positive, noting that the mentees seemed to support each other in their group meetings and felt quieter graduates may benefit from the presence of their peers. Mentor 1 commented that one-to-one meetings are more confidential, however confidentiality was not raised as a concern from one of the mentees who had attended the group meetings:

‘we could always be open and honest with each other and raise any concerns and if [the mentor] had any problems she wanted to raise she could obviously let me know.’ – *Mentee 1*

Similarly, this preference for group preceptorship was identified in a focus group of new graduate midwives (Mason and Davies 2013). Mentor 1 liked that the one-to-one sessions helped to build rapport between the mentee and senior staff member. This was mirrored in a comment made by mentee 2:

‘[the meetings] did help get a relationship with someone senior to yourself… I’m still able to talk to my preceptorship mentor quite comfortably… it’s nice to… build your confidence with a senior and also be able to build that relationship so you’re easily able to go to someone for help … and you’re quite comfortable talking to them about certain things I feel, and it does help in that sense, in building a relationship like that through work…’ – *Mentee 2*

Mentor 2 noted that she would worry a one-to-one meeting may feel more confrontational. Mentor 1 preferred the setting of a private meeting to give the mentee an opportunity to raise any confidential issues. She was however interested in the dynamic mentor 2 had achieved with having two graduates at once and considered trying group meetings for her current new graduates, who had just started the preceptorship.

#### Preceptorship structure

The Preceptorship document was structured by behaviour chapters where the mentee was required to document their evidence for each behaviour throughout the year. It also included a planner to co-ordinate meetings with the mentor.

Beecroft (2006) reported programme structure to be highly relevant. Regular meetings were significantly more likely to result in reports of better guidance, support, and stress relief (p < 0.001).

It was unanimously agreed that the structure of the preceptorship programme contributed to motivation and ease of transition for both mentees and mentors:

‘I really like it because it gives structure and it meant that we were meeting every couple of months. With previous new graduates… you make an effort for a one-to-one, but you don’t always get the opportunity’ – *Mentor 1*

‘I think for a less motivated new graduate… the very regular meetings… would help to remind them I think of the kind of things they should be considering.’ – *Mentor 2*

‘the structure really helps to make things manageable within the first year of work’ – *Mentee 3*

‘Having a structured meeting every month kind of helped and being able to meet up with someone and talk through what you’ve been going through… It kind of helped me in a sense to slowly get into it…’ – *Mentee 2*

Mentor 1 described the document as ‘invaluable’ as it allowed her to identify if her mentee was struggling with any aspect of her transition into autonomy. Mentor 2 mentioned how she had used her trust’s preceptorship prior to the release of the BIOS programme but had found it ‘nurse-oriented’ and ‘wasn’t of much relevance.’ She explained the BIOS programme seemed more ‘modern’ and ‘contextual’ in comparison.

### Barriers to the programme

While not identified as barriers at the time, participants discussed aspects of the programme that were more difficult to engage with, such as document navigation. Undergraduate and departmental engagement was also highlighted as something that could be improved to ensure more successful uptake and completion of the BIOS preceptorship.

#### Document navigation

As the BIOS preceptorship was newly developed, both mentees and mentors were new to the programme and it was described as a ‘learning curve’ by mentee 1 for both parties. Mentors initially did not find the document ‘user-friendly’:

‘I was a bit perplexed as how to use it because… it gives a bit of an explanation at the start but then it launches into finding examples.’ – *Mentor 1*

‘I think I find that there is quite a lot of overlap in some of the sections… the [mentees] weren’t very sure what would fit into which section.’ – *Mentor 2*

The confusion felt by mentor 1 when starting the document led her to complete a separate preceptorship e-learning course where she created her own supporting document which guided her to perform the role of preceptor more effectively. Both mentors agreed a supporting guidance document may be beneficial for mentors to understand how to fulfil their role as a BIOS preceptor. These findings are in-keeping with recent literature such as Maringer (2014), who concluded staff would require training in order to successfully fulfil the role of the preceptor. Clipper and Cherry (2015) also identified a higher retention rate in new graduate nurses who had trained preceptors compared to nurses who had untrained preceptors. Williams et al. (2018) found preceptors who had formal training in leadership were more confident in engaging in leadership activities with pharmacy students than those who did not receive training. This dictates specific training will contribute to better mentor engagement and consequently a more positive preceptorship experience for the new graduate. Higher perceived preceptor effectiveness has been shown to lead to psychological empowerment and professional autonomy in newly qualified nurses (Watkins, Hart, and Mareno. 2016). The perceived value of the BIOS preceptorship programme could be enhanced if mentors received guidance/training so that they felt confident in their delivery of the programme.

The contents of the document were highlighted as lacking in areas such as Continued Professional Development (CPD) and how to handle and report safeguarding cases where a new graduate may suspect the safety or wellbeing of a patient is compromised.

‘[a section] about examples of how to record my CPD because I really didn’t know to be honest… I appreciate again people have different styles, but… if you were picked for [the HCPC CPD audit], what would be expected in that case… it would just be nice to see what people would have wanted….’ –*Mentee 3*

‘Yeah, I agree… having little examples in there or even links to certain things that would be good as a resource would definitely help, because CPD was one thing I didn’t know how to like … because you don’t get much about it at uni I feel, you get like a little bracket of “this is what you have to do, make sure you note it down,” and then that was it, you kind of move on.’ – *Mentee 2*

‘I appreciate we have training and I’ve been on courses here since I’ve started, but I think an aspect of [safeguarding] would have been nice, just to say “be aware of this” in your first year …and then in that moment you’re not necessarily as supported.’ – *Mentee 3*

However, there was disagreement from the mentors;

‘[Safeguarding] is one of those things that you just have to do, to get the experience, you know to guide them through it.…if we’re safeguarding, anything that’s difficult the band 5 would escalate … So that’s why I’d struggle whether it needs to be in a preceptorship document.’ *– Mentor 1*

The mentors suggested a section on writing of procedures and departmental policies would be more appropriate to ensure mentees gained experience in upholding and developing local clinical governance.

During discussions on how the document could be improved, the general consensus was that each evidence chapter would benefit from having some examples of what to include, so mentees could understand what sort of skills the chapter was asking for and avoid repetition across chapters.

Areas that were flagged as being more difficult to evidence due to lack of examples were leadership and management, risk management, advocacy, and equality and diversity. Advocacy was noted as a difficult skill to evidence by both mentees and mentors as they struggled to think of examples for this term. It was agreed the contextual definition of advocacy was not clear. Similarly, Williams et al. (2018) found preceptors were least confident engaging students in advocacy activities. Williams suggests the reason for this may be that students do not consider advocacy as a priority learning experience. This could apply to the findings in this study; however, our study may indicate a lack of priority may come about when there is lack of contextual understanding.

Mentor 1 noted that her mentee may have overcomplicated some of the evidence chapters and even downplayed her ability due to the lack of examples, in particular the leadership and management chapter, and advised her to ‘look at [the chapter] from other perspectives’ and was able to suggest band-appropriate examples of leadership and management, such as caseload management and co-ordination of healthcare assistants. This downplaying behaviour has been reported on in the literature; Morley et al (2007) reported that new graduates often have a high expectation of their clinical competence and can therefore lead to graduates believing they are not as competent as they might actually be. It would be beneficial for mentors to be aware of this trait in order to encourage mentees to avoid it.

While the audit section was met with enjoyment from mentee 1, the mentors felt there was too much ‘superfluous information’ in this chapter and mentees could more easily be directed to audit resources from BIOS, to make it more ‘streamlined’ like other chapters. Mentor 1 pointed out audits may be carried out slightly differently across Trusts and therefore a strict template for completing an audit during the preceptorship might not match up with how a mentee’s Trust would expect an audit to be carried out.

#### Undergraduate/departmental engagement

There was consensus between both mentors and mentees that equal engagement from both parties is more likely to result in successful completion of the BIOS Preceptorship. Additionally, both groups agreed the promotion of the programme to departments and third year orthoptic students could be improved.

‘I feel if I knew about [the preceptorship] prior to [working]… I would have probably brought it up maybe at the interview, maybe once I started the job … that way I feel like you’re proactive in your own development as well. I feel like quite a few people would probably be more proactive if they knew about [the preceptorship] prior to starting their job. So maybe even having it mentioned at uni to students.’ – *Mentee 2*

‘I think there’s a mentality in some orthoptic departments… they just come to work and they go home… I think drilling it in at the [Leads of Orthoptic Profession] meetings is an important thing to get heads involved and then maybe that will filter down to new grads.’ – *Mentee 1*

This is reflected in the literature by Giallonardo, Wong and Iwasiw (2010) who explored leadership in preceptorship and concluded a mentor’s engagement and authenticity of leadership positively impacted new graduate nurses’ work management.

It was apparent the mentees in this study did experience a good level of engagement from their mentors. It may also be relevant however, to consider how engagement of a new graduate’s whole department can impact their transition experience. Departmental investment, similar to that which is granted by the BIOS preceptorship, has been identified as a key aspect that contributes to an effective preceptorship and a new graduate’s feeling of being an independent practitioner (Kaviani and Stillwell. 2000; Furness et al. 2020). A further study found significant stress-factors for new graduates to include encountering unhappy or unwilling staff (Oermann and Gavin 2002).

Furthermore, the mentors noted a barrier to engagement may be the misconception within departments that the preceptorship mentor needs to be the head of the department who may already have a heavy workload.

‘it doesn’t have to be someone who’s been qualified a long, long time or has a very senior position in the department, and if that was the case then maybe more departments would use it … because then it would be easier than taking a manager’s time.’ – *Mentor 2*

A further finding of Beecroft’s study (2006) indicated an older mentor resulted in significantly reduced feelings of stress in new graduates. This conflicts slightly with our participants’ feelings that the preceptorship could be carried out by any experienced orthoptist in order to avoid taking up more of a manager’s time. With Beecroft’s findings in mind, a non-managerial orthoptist but who has significant experience could positively impact preceptorship delivery and departmental uptake of the programme.

### Professional development

Autonomous behaviours, such as reflection and clinical governance were highlighted as areas the preceptorship actively promoted and contributed to the development of the new graduate to become an independent practitioner.

#### Reflection

The mentees discussed how the preceptorship had impacted their practice and clinical governance. They agreed the document promoted reflection, either by actively going through the chapters and answering the reflection questions, or by looking at the booklet as a whole and realising how much one covered in the first year of working.

‘I still [reflect] now, it definitely helped in that sense… you’re able to carry it on…’ – *Mentee 2*

There was consensus with the mentors, who agreed the document was focused on reflection, and noted this was an essential skill for effective clinical practice throughout one’s career.

‘Personally, I feel it’s good for me because I’ve gone and revisited things and provided teaching for them… to sit down and explain the difference between audit and research… it makes you realise how much experience you have yourself!’ – *Mentor 1*

The importance of reflection during preceptorship is abundant throughout the literature, with preceptorship in nursing and occupational therapy also identifying reflection as a cornerstone to success (Maringer 2014; Marks-Maran 2013).

#### CPD and Clinical governance

All mentees kept paper copies of their preceptorship document. Mentee 2 stated she found it a useful addition to her CPD portfolio. Mentor 1 mirrored this comment from her perspective:

‘I used [the preceptorship] as a basis of telling them the importance of maintaining a record of CPD and the need for a personal development plan…’ – *Mentor 1*

It was agreed valuable areas of clinical governance had been addressed in the programme.

‘… it really brought things to sort of focus and gave it structure. And I think … it made them go off and do things, like an audit or think about research… it just sort of gave it a place within the first year of qualification and … it just gave the opportunity to bring in lots of other things, got them looking at [standard operating procedures] and guidelines and things like that.’ – *Mentor 2*

#### Career progression

It is common for band 5 orthoptists to progress to band 6 after 12 months, and the mentors discussed how the preceptorship was a useful tool in this type of career progression for early career orthoptists.

‘…I want to get [the new graduates] up to speed because I’d like to progress them into the band 6 that I’m missing. You know so along with preceptorship I’ve got a list of you know all the competencies and experience that they need, and so it all feeds together and… then I’ve got that evidence that I can then take to panel to show, right, you know they’re ready for the band 6.’ – *Mentor 1*

#### Transition

The preceptorship programme aims to support the mentee in their transition from student to autonomous practitioner by asking the graduate to record and reflect on behaviours that contribute to that transition. Mentees described situations when they had demonstrated transitional behaviours during their preceptorship, such as leadership and management.

‘Over these last few months, we’ve had some new band 5 starters, so at that point then I got more examples for my leadership and management section of the booklet because I could show them things within our hospital, which otherwise if they weren’t here, I maybe wouldn’t have had the examples that I do now have.’ *– Mentee 3*

‘I think my [leadership] examples came from students that we had.’ – *Mentee 2*

‘I felt like I still was a student at first, so seeing these students I felt a bit odd…it took a while to understand that… I guess I do know a bit more than [the students]!’ *– Mentee 2*

The preceptorship had given mentee 3 the transitional skills to take initiative that benefitted her whole department:

‘I’ve made a healthy conversations and signposting quiz which particularly helped our band 5 new starters… because I am from [removed for anonymity] but work in [removed for anonymity], I don’t know what sort of support services are around here, so I thought it would be a nice idea to sort of explore what is round here for helping stopping smoking and alcoholism and support groups around here, and I’ve put things together within a nice document, which has been useful for everybody within the department…’ – *Mentee 3*

### Experience of Public Health during Preceptorship

The preceptorship aims to promote Public Health and encourage clinical behaviours that will contribute to Public Health campaigns, such as Make Every Contact Count (MECC) and healthy conversations. The participants discussed their experience with this campaign and how the preceptorship contributed to this experience.

The document specifically asks mentees to record their healthy conversations as a script, which was praised by the mentees:

‘…because I’d detailed [the conversation] as the script, and I could look back and think, yeah, maybe I’d change this, and after reading the top tips… on making every contact count, I sort of adapted my approach to what I could do better in future…’*– Mentee 3*

‘…my thought process changed about make every contact count, it’s not about telling a patient that they need to do something, it’s about making them aware of the impact of what they are doing is having. So, I think that quickly made it a bit easier for me…’ – *Mentee 1*

‘When you first graduate it is more difficult to have [healthy conversations]. But I quite liked that section of the booklet because it did make me think about the importance of having those conversations and … how I could structure them and how to say it a bit nicer to [the patients].’ – *Mentee 1*

While the Healthy Conversation chapter had been educational, participants were able to identify barriers that continued to impact their ability to have healthy conversations despite the aid of the preceptorship.

‘I would just say the other thing which is probably not ideal… the time aspect of when you’re at work and the pressure and seeing everybody, trying to stick to the time and you know you’ve got people waiting and it’s difficult to then start [the healthy conversation]…’ – *Mentee 3*

Our findings are similar to the themes reported in a focus group study exploring healthcare students’ perceptions about their role to deliver brief public health interventions (McLean et al. 2018) where the main barriers included lack of confidence and knowledge, time and the environment of the clinical placement.

Mentee 3 noted a barrier for her was the type of patients she would see day to day as, for example, she didn’t see many thyroid patients, making opportunities for healthy conversations about smoking rare. This conception that healthy conversations are only relevant to particular conditions needs further exploration and education to ensure health professionals are taking a holistic approach to public health.

Lawrence et al. (2016) found specific Healthy Conversation Skills training resulted in significantly increased use of these skills compared to untrained equivalents up to one year after training. While the BIOS preceptorship may offer useful reflection tools for Healthy Conversations, it is not designed to offer training as intense as what was implemented by Lawrence et al. due to its supplementary nature. The new graduates in Lawrence’s study may also have lacked the undergraduate education of the importance of public health as the recommendations for embedding public health education into the undergraduate curriculum was introduced in 2017 (Council of Deans 2017). Therefore, graduates of 2018 are more likely to have public health knowledge embedded in their curricula. The mentors suggested incorporating BIOS resources into undergraduate lectures as a form of Healthy Conversation training, such as MECC roleplay scenes found on the BIOS Youtube channel. The mentees explained they would feel more comfortable with healthy conversations if the patients knew to expect questions about lifestyle choices at their appointments. BIOS offer MECC posters to display in waiting rooms to pre-empt the conversations with the clinician about lifestyles and signposting. The mentees agreed having the posters in the waiting areas would improve their engagement in healthy conversations.

### Limitations

The advert received a low response rate with nine volunteers coming forward and five being available to attend the online interviews. Investigating the number of departments that utilised the preceptorship document may have offered complementary information and reasoning behind the low response rate, however as this information did not align with the aims of the project, this information was not gathered. No volunteers came forward who had completed a non-BIOS/no support mechanism in their first year, which limited the ability to compare the BIOS preceptorship to a control group and to other support mechanisms. This may have impacted the representativeness of the study and resulted in response bias as mentors/mentees who did not have a positive experience with their support tool/did not engage in any support tool may have felt less comfortable about volunteering. Furthermore, response bias may have also been a factor for those who did participate as the small group may have been at risk of acquiescence and desirability bias. Participants may have been tempted to report more positive and desirable experiences in order to make themselves and their preceptorship experience appear more favourable. The interviewers minimised this risk by encouraging openness and honesty without judgement at the beginning of the interviews, however it is still acknowledged as a potential limitation.

## Conclusion

The preceptorship programme by BIOS was successful in engaging new graduate orthoptists in constructive professional behaviours which contributed to career progression and helped to harbour a positive relationship between mentee and mentor which continued post-preceptorship. The interviews demonstrated the BIOS preceptorship successfully embedding behaviours that contribute to high quality practice in healthcare, such as leadership and autonomy. In order for a preceptorship to be successful, engagement from both preceptee and preceptor is necessary.

In order to improve engagement in the BIOS preceptorship programme, we recommend a drive to inform third year orthoptic students of its benefits and what to expect when starting the preceptorship. The document itself can also be distributed to leads of service for dissemination among departments to ensure employers are also aware of the preceptorship and its benefits. There may be departmental misconceptions that the responsibility of mentor falls to the department head. We suggest an orthoptist who feels confident in their own level of experience for each of the preceptorship chapters would be an appropriate choice for preceptor. However, a key finding from this study was the need for mentor guidance/training in preparation for the preceptor role. We highly recommend every clinician who is nominated for the role of preceptor receive formal guidance/training, either in the form of a guidance booklet for the mentor to refer to during the programme, or a preceptorship course prior to the programme.

The BIOS Preceptorship was also seen to encourage Public Health behaviours and attitudes through reflection on healthy conversations. Lack of confidence, general public’s knowledge of Public Health and clinical time pressures were identified as barriers to embedding Public Health into clinical practice. Suggestions to improve engagement in Healthy Conversations and the MECC campaign include increasing the amount of undergraduate experience of healthy conversations, potentially by means of specific Healthy Conversation skills training and engagement on clinical placement. We suggest any modifications made to future versions of the preceptorship should include these barriers as a discussion point, so even if they are still present, new graduates can be aware of them and attempt to overcome them where possible.
